# Efferocytosis and Its Role in Inflammatory Disorders

**DOI:** 10.3389/fcell.2022.839248

**Published:** 2022-02-25

**Authors:** Yun Ge, Man Huang, Yong-ming Yao

**Affiliations:** ^1^ Department of General Intensive Care Unit, The Second Affiliated Hospital of Zhejiang University School of Medicine, Hangzhou, China; ^2^ Translational Medicine Research Center, Medical Innovation Research Division and Fourth Medical Center of the Chinese PLA General Hospital, Beijing, China

**Keywords:** apoptosis, efferocytosis, inflammatory diseases, immune response, phagocytosis

## Abstract

Efferocytosis is the effective clearance of apoptotic cells by professional and non-professional phagocytes. The process is mechanically different from other forms of phagocytosis and involves the localization, binding, internalization, and degradation of apoptotic cells. Defective efferocytosis has been demonstrated to associate with the pathogenesis of various inflammatory disorders. In the current review, we summarize recent findings with regard to efferocytosis networks and discuss the relationship between efferocytosis and different immune cell populations, as well as describe how efferocytosis helps resolve inflammatory response and modulate immune balance. Our knowledge so far about efferocytosis suggests that it may be a useful target in the treatment of numerous inflammatory diseases.

## Introduction

Cell turnover is usually achieved through apoptosis (Elmore, 2007) and other newly regulated cell death programs. Importantly, dying cells release molecular signals that direct phagocytes to sites of death and regulate immune response to maintain tissue homeostasis (Devitt and Marshall, 2011). This multi-step process is known as efferocytosis, which comes from the Latin word “effere”, meaning “take to the grave” (de Cathelineau and Henson, 2003). In the pathogenesis of efferocytosis, phagocytes such as macrophages, dendritic cells (DCs), monocytes, and epithelial cells destroy and recycle dead cells (Arandjelovic and Ravichandran, 2015). In fact, so-called “find me” signals recruit phagocytes, while “eat me” signals trigger uptake of apoptotic cells.

Accumulating evidence suggests that efferocytosis is vital for tissue repair, inflammation resolution, and immune system balance during homeostasis (Monks et al., 2005; Arandjelovic and Ravichandran, 2015; Sachet et al., 2017; Boada-Romero et al., 2020). Accordingly, impaired efferocytosis can lead to the accumulation of apoptotic cells in inflamed foci, subsequently resulting in cell necrosis, cytolysis, and the production of tissue-damaging intracellular components (Monks et al., 2005; Szondy et al., 2014; Arandjelovic and Ravichandran, 2015; Sachet et al., 2017). Moreover, abnormal efferocytosis may induce substantial inflammatory response and contribute to the development of various inflammatory disorders (McCubbrey and Curtis, 2013; Karaji and Sattentau, 2017; Abdolmaleki et al., 2018; Horst et al., 2019; Morioka et al., 2019; Doran et al., 2020; Yoshimura et al., 2020). Over recent years, the underlying mechanisms and regulatory pathways of efferocytosis in inflammatory and autoimmune diseases have been widely studied. This review summarizes the recent findings with regard to efferocytosis signals and their impacts on host immunity, which might be of importance to understand the pathophysiology of abnormal efferocytosis and inflammatory diseases.

## Efferocytosis and Its Receptor Network

**FIGURE 1:**
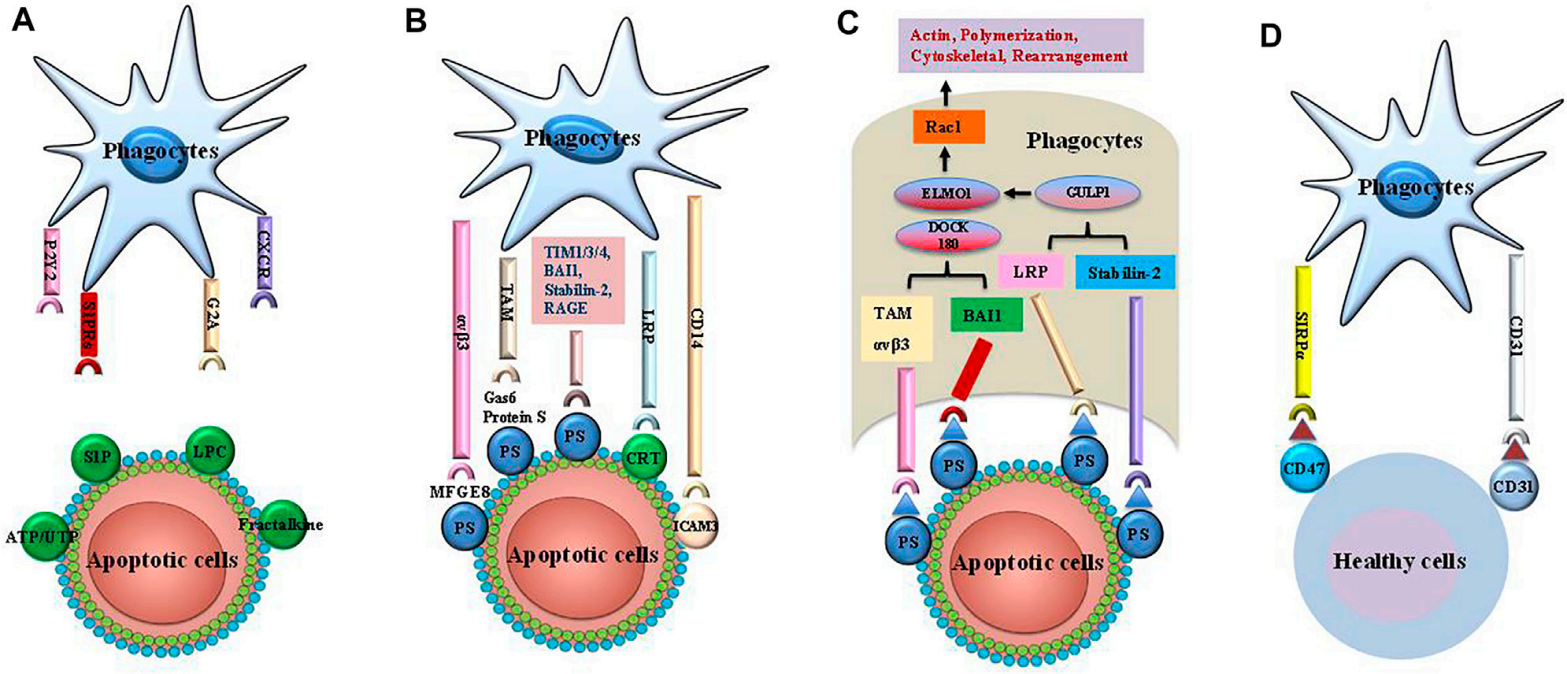
The steps of efferocytosis. Efferocytosis is a multi-steps process that involves several steps: finding apoptotic cells, binding apoptotic cells, internalizing and digestion of apoptotic cells. **(A)** The apoptotic cells release a series of “find me” signals including lysophosphatidylcholine sphingosine-1-phosphate (S1P), uridine diphosphate (UTP) and adenosine triphosphate (ATP), which attract phagocytes to region of apoptotic corpses. These signals are recognized by phagocytes using cognate receptors such as CXCR3, G-protein-coupled receptor (G2A), purinergic receptors (P2Y2), andsphingosine-1-phosphate receptor (S1PRs). **(B)** The “eat me” signals on the apoptotic cells are sensed by phagocytes, which ingest these dying cells *via* several receptors and bridging molecules. These crucial signals comprise brain-specific angiogenesis inhibitor 1 (BAI1), T cell immunoglobulin mucin receptor (TIM) 1, TIM3, TIM4, the receptor for advanced glycationend products (RAGE), stabilin-2, phosphatidylserine (PS)-specific bridging molecules, growth arrest specific 6 (Gas6), milk fat globule epidermal growth factor VIII (MFG-E8), and protein S. In addition, calreticulin (CRT) and intercellular adhesion molecule (ICAM) 3 act as the “eat me” signals *via* interaction with CD14 and low-density lipoprotein-related protein (LRP). **(C)** Engulfment of apoptotic cells are conducted by phagocytes by recruitment of ingestion receptors along with Rac pathways, the polymerization of actin and rearranging of cytoskeletal. Ingestion receptors can recruit the DOCK180/ELMO1 set [αvβ3, integrin, Tyro3-Ax1-MER proto-oncogene tyrosine kinase (MERTK) (TAM), stabilin-2, and LRP]. D. Healthy cells can resist efferocytosis and leave phagocytes unengulfed via “tolerate me” signals (e.g., CD47, CD31) on the cell surface. SIRPα on the surface of phagocytes can recognize CD47. Similarly, CD31 homodimerizes with CD31 on the phagocytes. Finally, mitochondrial fission and an increase in cytoplasmic calcium occurs in efferocytes. These cellular changes are critical for phagosome sealing. Phagosome fusion with lysosome leads to degradation of apoptotic cells via acid hydrolase activity. The processing of engulfed apoptotic corpses uses microtubule-associated protein light chain 3 (LC3)-dependent phagocytosis and exhibit anti-inflammatory activities.

Apoptosis is a highly organized process that accelerates embryogenesis and maintains cell growth (Elmore, 2007). Generally, apoptosis is terminated by efferocytosis, which prevents the aggregation of cell corpses, inflammatory response, and secondary necrosis of other cells (Devitt and Marshall, 2011; Arandjelovic and Ravichandran, 2015). Efferocytosis is performed by professional phagocytes, including DCs and macrophages, and non-professional phagocytes, such as fibroblasts and epithelial cells, which recognize “find me” and “eat me” signals from apoptotic cells (Arandjelovic and Ravichandran, 2015). Specifically, efferocytosis involves four steps (Figure 1): 1) phagocyte recruitment regulated by “find me” signals, 2) identification of dead cells guided by “eat me” signals, 3) uptake of cellular corpses (Monks et al., 2005; Boada-Romero et al., 2020), 4) degradation of dying cells (Monks et al., 2005; Boada-Romero et al., 2020). Nevertheless, healthy cells can escape efferocytosis *via* “tolerate me” signals, also known as “keep me” or “don’t eat me” signals (Doran et al., 2020).

### “Find Me” Signals

“Find me” signals are released by apoptotic cells to distinguish them from healthy cells and to recruit phagocytes to sites of death (Ravichandran, 2010; Doran et al., 2020). These signals also act as danger-associated molecular patterns (DAMPs) and mediate the formation of various cytokines as well as chemokines for phagocyte activation (Karaji and Sattentau, 2017; Morioka et al., 2019). “Find me” signals consist of sphingosine-1-phosphate (S1P), lysophosphatidylcholine (LPC), C-X-C motif chemokine ligand 1 (CX3CL1), and nucleotides (Gude et al., 2008; Truman et al., 2008; Elliott et al., 2009; Chekeni et al., 2010; Garris et al., 2013). S1P is produced from sphingosine *via* sphingosine kinases and modulates phagocyte chemotaxis by engaging G-protein-coupled receptors (Gude et al., 2008; Garris et al., 2013), while LPC is generated by caspase-3 and phospholipase A_2_ (Ravichandran, 2010). Nucleotides such as uridine diphosphate and adenosine triphosphate (ATP) promote the interaction of phagocytes with purinergic receptors, thus favoring phagocytic clearance of apoptotic cells (Elliott et al., 2009). As a chemokine, CX3CL1 is released from apoptotic lymphocytes in a caspase- and Bcl-2-dependent manner. Macrophages are attracted to apoptotic sites via interaction with CX3CL1 and macrophage fractalkine receptor (a “find-me” signal) (Peter et al., 2008; Truman et al., 2008). However, the underlying molecular mechanisms are obscure.

### “Eat Me” Signals

In the second step of efferocytosis, apoptotic cells bind directly to cell surface receptors (e.g., low-density lipoprotein receptor-related protein 1, T cell immunoglobulin mucin receptor (TIM) 1, TIM3, TIM4, adhesion G protein-coupled receptor B1, stabilin-1, and stabilin-2) (Gardai et al., 2005; Park et al., 2008a; Rodriguez-Manzanet et al., 2010; He et al., 2011; Mazaheri et al., 2014), in turn inducing pleiotropic effects through several bridging molecules such as protein S, milk fat globule epidermal growth factor 8 (MFG-E8), and vitamin K-dependent protein growth arrest specific 6 (Gas6) (Fadok et al., 1998a; Scott et al., 2001; Thorp et al., 2008; Hu et al., 2009; Qi et al., 2013; Nguyen et al., 2014; Soki et al., 2014; Toda et al., 2014; Nepal et al., 2019; Rymut et al., 2020). Similarly, transglutaminase 2 (TG2) acts as a coreceptor for integrin β_3_ and binds MFG-E8, inducing uptake of apoptotic cells via activating Rac 1. Conversely, integrin β_3_ cannot recognize the apoptotic cells in the absence of TG2 (Tóth et al., 2009; Sághy et al., 2019).

Phosphatidylserine (PS), which is found in the inner leaflet of living cells and is expressed externally *via* caspase signals during apoptosis (Park et al., 2008b; Segawa et al., 2014), appears to be a key factor in “eat me” signals. “Eat me” signals are recognized directly by PS binding receptors or indirectly by bridging mediators on phagocytes. Direct binding of PS to the receptor augments the formation of advanced glycation end products (RAGE) and macrophage efferocytosis (Thorp et al., 2008; Birge et al., 2016). MFG-E8 is capable of recognizing PS and being recognized by phagocyte (e.g., DCs, macrophages) surface receptors αVβ3 and αVβ5. Interaction with these receptors can lead to cytoskeletal rearrangements and then promote the uptake of apoptotic cells (Hanayama et al., 2004; Hu et al., 2009; Soki et al., 2014). In addition, bridging of the complement factor C1q with PS is recognized by scavenger receptor class F member 1 on endothelial and phagocytotic cells (Pulanco et al., 2017), while soluble CD93 interacting with PS and integrin αxβ2 on apoptotic cells mediates efferocytosis *via* an opsonin (Martin et al., 1996). These results suggest that interaction with PS can accelerate the engulfment of dying cells.

PS serves as the most characterized “eat-me” signal. PS can be recognized by several membrane receptors such as Stabilin-1, Stabilin-2, TIM4, RAGE, and BAI-1. It has been demonstrated that PS receptors play a key role in the recognition mechanism of dead cells (Park et al., 2008a; Rodriguez-Manzanet et al., 2010; Mazaheri et al., 2014; Nishi et al., 2014). For example, Stabilin-1 and -2 expressed by macrophages recognize PS on apoptotic cells and enhance the ingestion of apoptotic debris (Park et al., 2008b; Rantakari et al., 2016). This process is essential for the capture and elimination of PS-stimulated injured or aged erythrocytes. The CD300 family of type I transmembrane proteins can recognize PS and phosphatidylethanolamine during apoptosis (Voss et al., 2015). Thus, CD300f and CD300d deficiency can disrupt efferocytosis by macrophages (Tian et al., 2016). The scavenger receptors SR-A1, SR-B1, and CD36 recognize PS and promote efferocytosis by macrophages (Fadok et al., 1998b; Terpstra and van Berkel, 2000).

As we known, high mobility group box-1 protein (HMGB1) is a classical DAMP that can suppress RAGE/PS-mediated efferocytosis by binding to integrin αvβ3 in macrophages (Friggeri et l., 2010). Conversely, HMGB1-deficient macrophages effectively phagocytose apoptotic neutrophils and thymocytes (Wang et al., 2013), leading to translocation of HMGB1 into the cytoplasm and its secretion into the extracellular milieu (Banerjee et al., 2011). In addition, the Ras homolog family (Rho) of small GTPases, including Rho-associated coiled-coil-containing protein serine/threonine kinase, Rho A, CDC42, Rab5, and Rac, is critically involved in regulating the uptake of dying cells (Castellano et al., 2000; Erwig et al., 2006; Nakaya et al., 2006; Moon et al., 2010).

### Uptake and Degradation of Dying Cells

Phagocytes recognize and home to cell corpses, then internalize them *via* plasma membrane reorganization (Boada-Romero et al., 2020). The remodeling of actin leads to invagination and localized extravagation of the plasma membrane and phagosome formation. After cell engulfment, the resulting phagosome fuses with lysosomes to digest cell corpses (Boada-Romero et al., 2020; Doran et al., 2020). Lysosomes contain several lipases, nucleases, and proteases that digest the apoptotic cells to maintain homeostasis (Viaud et al., 2018). Microtubule-associated protein light chain 3 is involved in the canonical autophagy pathway, while unc-51-like kinase 1/2 complex plays a crucial role in the digestion of dying cells (Florey et al., 2011; Asare et al., 2020).

### “Tolerate Me” Signals

Healthy cells express transmembrane molecules that down-regulate efferocytosis through “tolerate me” signals (Martinez, 2017). Binding of CD47 to the signal regulatory protein-α on the macrophage surface inhibits the actin cytoskeleton rearrangements required for phagocytosis (Willingham et al., 2012; Kojima et al., 2016; Cao et al., 2022). Phagocytic clearance is blocked by the sialoglycoprotein CD24, which binds to sialic acid-binding Ig like lectin 10 (Siglec-10) on macrophages (Barkal et al., 2019). CD31 on macrophages and healthy cells can inhibit phagocytosis (Brown et al., 2002).

Major histocompatibility complex (MHC) class I molecules are positively expressed on healthy cells and interact with the inhibitory receptor leukocyte immunoglobulin-like receptor subfamily B member 1, which contribute to blocking the engulfment of apoptotic cells and the expression of inflammatory molecules (Barkal et al., 2018). However, the release of lactoferrin glycoprotein from apoptotic cells acts as a “tolerate me” signal, in turn removing eosinophils and neutrophils from the sites of death (Bournazou et al., 2009; Bournazou et al., 2010). Recently, plasminogen activator inhibitor-1 has been shown to induce excessive accumulation of neutrophils and inflammatory response in tissues, leading to organ dysfunction. Therefore, it may be a novel “tolerate me” signal for apoptotic neutrophils (Park et al., 2008a).

## Efferocytosis in Immune Cells and Underlying Regulatory Mechanisms

### Efferocytosis and Macrophages

Macrophages play important roles in innate immunity and the restoration of tissue homeostasis, as they ingest infected apoptotic cells to kill bacteria and limit cell necrosis, while they drive adaptive immune response by degrading pathogen-associated antigens and presenting them to effector T cells (Martin et al., 2014; Blander, 2017).

Macrophages can exhibit two main phenotypes depending on the stimuli and microenvironment: a pro-inflammatory M1 phenotype, or a pro-resolving M2 (“alternatively activated”) phenotype (Angsana et al., 2016; Elliott et al., 2017). Efferocytosis shifts macrophages towards the M2 phenotype, which can reduce levels of pro-inflammatory cytokines [e.g., tumor necrosis factor alpha (TNF-α), CXCL-8, LBT4, interleukin (IL)-6] and enhance the release of anti-inflammatory mediators [IL-10 and transforming growth factor-beta (TGF-β)] as well as pro-resolving molecules (Fadok et al., 1998a; Dalli and Serhan, 2012; Angsana et al., 2016). The resolution of inflammation is regulated by the balance between pro-inflammatory cytokines and pro-resolving mediators, such as lipoxins and resolvins, which have been shown to augment efferocytosis of apoptotic cells by macrophages (Korns et al., 2011; Schif-Zuck et al., 2011). For instance, resolvin E4 effectively limits neutrophil infiltration and induces efferocytotic ingestion of apoptotic neutrophils and senescent red blood cells by macrophages. Likely, resolvin D1 improves efferocytosis in aging by limiting senescent cell-mediated MERTK cleavage (Schif-Zuck et al., 2011; Nishi et al., 2014). MERTK-binding bridging molecules contribute to efferocytosis-associated inflammatory resolution (Nishi et al., 2014; Toda et al., 2014), while the Tyro3-Ax1-MERTK (TAM) family of receptor tyrosine kinases in macrophages is involved in the recognition and clearance of dead cells, as well as the activation of anti-inflammatory signals (Qi et al., 2013; Toda et al., 2014; Bernsmeier et al., 2015). Furthermore, efferocytosis can promote non-inflammatory macrophage proliferation to help resolve tissue injury *via* inducing DNA-dependent protein kinase-mammalian target of rapamycin-rictor complex 2/Rictor pathway (Gerlach et al., 2021).

Efferocytosis is an anti-inflammatory process sensitive to cyclic adenosine 3,5′-monophosphate (cAMP). cAMP is a crucial intracellular molecule that affects phagocytosis and reprogramming of macrophages for inflammation resolution, while it can stimulate macrophage efferocytosis of apoptotic neutrophils by protein kinase A (PKA) signaling (Negreiros-Lima et al., 2020). In addition, the secreted protein endothelial locus-1 (DEL-1) has recently been found to inhibit leukocyte-endothelial adhesion and inflammation initiation, serving as a potent stimulator of efferocytosis (Kourtzelis et al., 2019). Moreover, DEL-1-mediated efferocytosis reprograms macrophages to adopt the pro-resolving phenotype, suggesting that DEL-1 facilitates homeostatic functions in the setting of inflammation (Kourtzelis et al., 2019).

### Efferocytosis and Dendritic Cells

It is widely accepted that DCs are professional antigen-presenting cells that can activate T cells. Costimulatory molecules, including CD80, CD86, and MHC-II, are crucial for DC maturation and subsequent activation of native CD4^+^ T cells, and their deficiency contributes to T cell immunosuppression or tolerance (Blachere et al., 2005). DCs have been identified as key phagocytes in recognizing apoptotic cells and regulating adaptive immunity (Albert et al., 1998; Schaible et al., 2003; Blachere et al., 2005), while they efficiently mediate efferocytosis to suppress immune response to self-antigens. Moreover, phagocytosis of infected apoptotic cells by DCs releases high amounts of CD86 and CC-chemokine receptor type 7 and favors the production of prostaglandin E_2_ (PGE_2_), IL-10, and IL-1β (Pujol-Autonell et al., 2013; Dejani et al., 2018). In contrast, inhibiting the recognition of infected cells markedly prevent the maturation of DCs (de Aquino Penteado et al., 2017). Efferocytosis of sterile apoptotic cells only slightly affects the phenotype and immune properties of DCs (de Aquino Penteado et al., 2017), yet it acts *via* PGE_2_ to down-regulate host immunity to self-antigens (Pujol-Autonell et al., 2013). Of note, further studies should be performed to investigate the opposing impacts of efferocytosis on DC maturation.

Several studies have shown that efferocytosis is involved in the cross-presentation and activation of CD8^+^ and CD4^+^ T cells during viral infection and tumor growth (Yrlid and Wick, 2000; Larsson et al., 2002; Bosnjak et al., 2005; Desch et al., 2011; Tzelepis et al., 2015). DCs recognize and ingest infected apoptotic cells, thus increasing the immune response to invading organisms (Albert et al., 1998; Blachere et al., 2005). Efferocytosis of infected apoptotic cells improved the production of IL-6 and PGE_2_, and the expression of CCR7 and CD86, and migration on DCs. Following recognition of infected cells, the maturation and migration of DCs correlated with high expression of cyclooxygenase-2 (COX-2) and PGE_2_, and activation of pattern recognition receptors by bacterial components (Yrlid and Wick, 2000; Pujol-Autonell et al., 2013; Tzelepis et al., 2015; Dejani et al., 2018), which further promoted lymph node-directed migration and up-regulated a Th2-type immune response (Dejani et al., 2018). Similar results were observed for apoptotic cells infected with influenza virus, human immunodeficiency virus (HIV)-1, herpes simplex virus, *Salmonella typhimurium*, *Mycobacterium tuberculosis,* vaccinia virus, and human cytomegalovirus (Larsson et al., 2002; Bosnjak et al., 2005; Desch et al., 2011; Tzelepis et al., 2015). For example, HIV-1-infected dying monocytes were phagocytosed by DCs, resulting in antigen cross-presentation (MHC class I or II) and T cell activation (Larsson et al., 2002; Werfel and Cook, 2018). Collectively, the available data indicate that efferocytosis of infected apoptotic cells by DCs leads to antigen presentation and activation of effector T cells together with elevation of COX-2 and PGE_2_ levels.

### Efferocytosis and Neutrophils

Neutrophils are short-lived cells that act as first responders of innate immunity and infiltrate into inflamed sites upon infection (Greenlee-Wacker, 2016). However, substantial neutrophil aggregation and secondary necrosis exacerbate inflammatory cascades, thereby leading to self-amplifying tissue injury and organ dysfunction (Greenlee-Wacker, 2016). Neutrophils can be inactivated *via* apoptosis, forming apoptotic cells that are removed by tissue-resident macrophages through an efferocytotic mechanism (Maimon et al., 2020; Sekheri et al., 2020). It was reported that culture of macrophages or monocytes with apoptotic neutrophils increased the levels of IL-10 and TGF-β (Kim et al., 2019), while their engulfment enhanced the release of specialized pro-resolving mediators, such as lipoxin B4 and resolvin 1/2, indicating the potential anti-inflammatory action of apoptotic neutrophils (Sun et al., 2015; Moges et al., 2018). Additionally, neutrophil efferocytosis guided by phagocytic signals was found to be regulated by urokinase receptor-associated procedures (Park et al., 2009).

There is increasing evidence that neutrophil function may affect efferocytotic activity in macrophages. Neutrophils exert a specific defense mechanism against microbes, known as neutrophil extracellular traps (NETs), which can capture and kill extracellular bacteria (Chen et al., 2021). NETs act through a unique form of non-apoptotic cell death, defined as NETosis, where molecules produced by neutrophils interfere with the recognition of dying cells (Bukong et al., 2018). For instance, HMGB1 serves as a DAMP that activates pro-inflammatory cascades [e.g., Toll-like-receptor (TLR), NLR family, pyrin domain-containing 3 (NLRP3)] and inhibits efferocytosis. In such process, HMGB1 binds to PS or RAGE receptors, thereby preventing the recognition of other PS-binding proteins (Friggeri et al., 2010; Banerjee et al., 2011; Wang et al., 2013).

Neutrophils modulate an efferocytotic “Trojan horse” way in certain infections. Bacteria such as *Yersinia pestis* and *Chlamydia pneumonia* can be phagocytosed by neutrophils, within which the bacteria multiply. Then neutrophils are engulfed by macrophages, promoting efferocytosis and increasing PS exposure (Jondle et al., 2018; Lim et al., 2020). Reportedly, phagocytosis of methicillin-resistant *Staphylococcus aureus* by neutrophils mediated the expression of the “tolerate me” signal CD47 and prevented macrophage efferocytosis (Willingham et al., 2012; Greenlee-Wacker et al., 2014). Failure to efferocytose dying and infected neutrophils resulted in neutrophil necrosis and the release of living bacteria (Lim et al., 2020). In line with these observations, *Klebsiella pneumoniae* escaped killing by macrophage-induced efferocytosis, as it prevented neutrophil pyroptosis and efferocytosis (Jondle et al., 2018).

### Efferocytosis and Regulatory T Cells

Regulatory T cells (Tregs) interact with various innate and adaptive immune cells and exert potent immunosuppression by inhibiting T cell function, promoting macrophages to the M2 phenotype, releasing anti-inflammatory mediators, enhancing immune tolerance, and accelerating inflammation resolution (Josefowicz et al., 2012; Weirather et al., 2014). Tregs have been found to modulate macrophage efferocytosis in animal models of inflammatory states, including peritonitis, atherosclerosis, and acute lung injury (ALI) (Proto et al., 2018). IL-13 secretion by Tregs stimulates the production of IL-10 in macrophages, which in turn induces macrophage efferocytosis by initiating Rac1-associated actin accumulation in the phagosome and apoptotic cell internalization (Proto et al., 2018), thus enhancing the proliferative activity of Tregs. The disposal of apoptotic cells leads to the production of IL-10 and TGF-β in macrophages, further increasing the Treg population (Kleinclauss et al., 2006). Similarly, apoptotic cell infusion accelerates the expansion of Tregs (Cummings et al., 2016). CD103^+^ DCs also engulf apoptotic cells, stimulating the differentiation of Tregs and inducing intestinal epithelial cell apoptosis [106]. Consistent with these results, phagocytosis of non-infected cells by macrophages favors the generation of Tregs and the production of anti-inflammatory mediators including PGE_2_, platelet-activating factor, and TGF-β (Tiemessen et al., 2007; Proto et al., 2018).

### Efferocytosis and Other Immune Cells

Ly6C^+^ monocytes were reported to induce efferocytosis *via* TLR ligation (Larson et al., 2016). For instance, TLR7 stimulated Ly6C^+^ monocytes to improve the cross-presentation of cell-associated antigen to CD8^+^ T cells, implicating a role for these monocytes in protective immunity (Arrode et al., 2000). Efferocytosis of infected apoptotic cells appeared to favor T helper (Th)17 immune response. Phagocytosis of *Escherichia coli*-infected cells by DCs facilitated the production of PGE_2_, IL-1β, and pro-Th17 cytokines, such as IL-6 and TGF-β (Torchinsky et al., 2009), while PGE_2_-EP4 signaling obviously inhibited Th17 cell differentiation and phosphorylation of signal transducer and activator of transcription (STAT) 3 (Monks et al., 2008). Strikingly, apoptotic epithelial cells were engulfed by residual viable epithelial cells into spacious efferosomes during post-lactation involution of the mouse mammary gland (Monks et al., 2005; Monks et al., 2008).

## Abnormal Efferocytosis and Inflammatory Disorders

**FIGURE 2:**
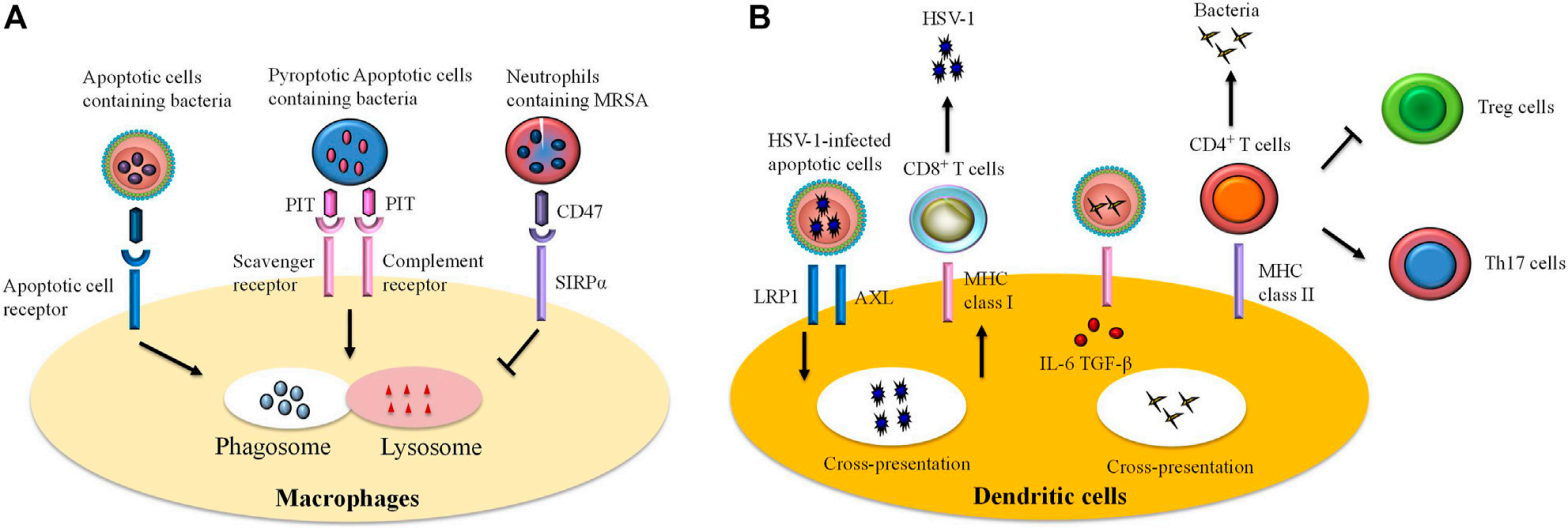
The role of efferocytosis by immune cells in infections. Efferocytosis participates in host defense against invading microbes. However, certain pathogens can escape efferocytosis and accelerate their spread. Efferocytosis is accomplished by professional and unprofessional phagocytes. Remarkably, macrophages and dendritic cells are professional phagocytes that are the most commonly studied of the efferocytes. **(A)** Ingestion of infected apoptotic cells by macrophages limits secondary necrosis of these cells and bacteria release, thereby improving bacteria clearance. In some cases, certain bacteria can trigger pyroptosis in infected cells. Efferocytotic receptors (i.e., scavenger receptor, complement receptor) are able to recognize pore-induced intracellular traps (PITs) and pytoptotic neutrophil containing bacteria. Subsequently, the process results in bacteria killing *via* infusion of phagosome to lysosome. However, some pathogens [e.g., methicillin-resistant *Staphylococcus aureus* (MRSA)] escape efferocytosis through “tolerate me” signal CD47 and signal regulatory protein α (SIRPα) on the infected cells. **(B)** Recognition and internalization of some viruses [such as herpes simplex virus type 1 (HSV-1)]-infected cells by dendritic cells interact with RAN-binding protein 9 (RANBP9), low-density lipoprotein receptor-related protein 1 (LRP1), and a protein complex comprising AXL. The cross presentation of viral antigen by dendritic cells on MHC class I molecules induces differentiation of CD8^+^ T cells against viruses. Similarly, dendritic cells swallow infected cells and thereby promote expansion of anti-bacteria effector T cells. In the infected cells, pathogen-associated molecular patterns (PAMPs) signal *via* Toll-like receptors (TLRs) and stimulate production of transforming growth factor-beta (TGF-β) and interleukin (IL)-6, thereby expanding the population of CD4^+^ T cells to T helper (Th)17 cells but inhibiting generation of regulatory T (Treg) cells.

**FIGURE 3:**
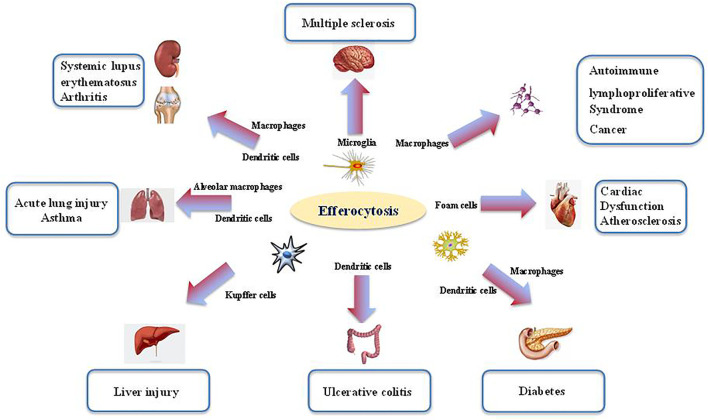
The role of efferocytosis in various inflammatory disorders. Efferocytosis is required for tissue homeostasis and organ development. The process is mainly orchestrated by several phagocytes (i.e., microglia, macrophage, dendritic cells). Aberrant efferocytosis has been associated with several inflammatory and autoimmune disorders, including infection, acute lung injury (ALI), asthma, systemic lupus erythematosus (SLE), rheumatoid arthritis (RA), diabetes, multiple sclerosis (MS), autoimmune lymphoproliferative syndrome (ALPS) and other inflammatory conditions.

Defects in efferocytosis has been demonstrated to result in substantial inflammatory responses that do not resolve, leading in turn to various pathologies (Morioka et al., 2019). Aberrant efferocytosis appears to be involved in several inflammatory and autoimmune disorders, including infection (Figure 2), ALI, asthma, systemic lupus erythematosus (SLE), rheumatoid arthritis (RA), diabetes, multiple sclerosis (MS), autoimmune lymphoproliferative syndrome (ALPS), and other inflammatory conditions (Figure 3 and Table 1) (McCubbrey and Curtis, 2013; Szondy et al., 2014; Karaji and Sattentau, 2017; Abdolmaleki et al., 2018; Horst et al., 2019; Doran et al., 2020; Yoshimura et al., 2020).

**TABLE 1:** Summary of studies concerning the significance of efferocytosis in various inflammatory diseases.

Diseases	Year	Authors	Observations or conclusions
Infection	2005	Bosnjak et al	Herpes simplex virus infection of human dendritic cells (DCs) induces apoptosis and allows cross-presentation via uninfected DCs
	2002	Larsson et al	The dead cells expressing HIV-1 antigens as well as non-infectious HIV-1 particles can be acquired and processed by DCs, leading to the activation, differentiation, and expansion of viral antigen-specific CD4 and CD8 T cells from seropositive individuals
	2015	Tzelepis et al	Annexin1 regulates DC efferocytosis and cross-presentation during *Mycobacterium tuberculosis* infection
	2018	Jondle et al	*Klebsiella pneumoniae* infection of murine neutrophils impairs efferocytic clearance by modulating cell death machinery
	2018	Codo Et al	Inhibition of inflammasome activation by a clinical strain of *Klebsiella pneumonia* impairs efferocytosis and leads to bacterial dissemination
	2002	Watanabe et al	Sugar chains are desialylated by neuraminidase on the surface of virus-infected cells. The presence of both phosphatidylserine and asialoglycomoieties on the cell surface is required for efficient phagocytosis of influenza virus-infected cells by macrophages
	2016	Cohen et al	*Staphylococcus aureus* down-regulates efferocytosis of neutrophils by macrophages through the activity of its virulence factor alpha toxin
	2020	Shibata et al	Respiratory syncytial virus infection exacerbates *Pneumococcal* pneumonia via Gas6/Axl-mediated macrophage polarization
	2021	dos-Santos et al	Efferocytosis of SARS-CoV-2-infected dying cells impairs macrophage anti-inflammatory programming and continual clearance of apoptotic cells
	2018	Grégoire et al	Macrophage engulfment of NETs and apoptotic neutrophils is diminished in ARDS patients. Notably, activation of AMPK in macrophages or neutralization of HMGB1 in BAL fluid improves efferocytosis and NET clearance
ALI	2022	Yan et al	Pentraxin 3 located on the membrane of apoptotic cells facilitates macrophage efferocytosis efficiently and alleviates lung inflammation in hard metal-induced acute lung injury
	2013	Juncadella et al	Apoptotic cell clearance by bronchial epithelial cells critically influences airway inflammation
Asthma	2019	Erriah et al	Galectin-3 enhances monocyte-derived macrophage efferocytosis of apoptotic granulocytes in asthma
SLE	2009	Hu et al	Genetic polymorphism in MFG-E8 is associated with SLE in human
	2004	Hanayama et al	Autoimmune disease and impaired uptake of apoptotic cells in MFG-E8-deficient mice
	2003	Potter et al	Lupus-prone mice have an abnormal response to thioglycolate and a dampened clearance of apoptotic cells
Arthritis	2018	Waterborg et al	Treatment of mice with MER receptor agonistic antibodies is deleterious due to its counterproductive effect of blocking efferocytosis in the arthritic joint
ALPS	2001	Bleesing et al	TcR-α/β^+^ CD4^−^ CD8^−^ T cells in humans with the autoimmune lymphoproliferative syndrome express a novel CD45 isoform that is analogous to murine B220 and represents a marker of altered O-glycan biosynthesis
Diabetes	2019	Luo et al	The deficiency of macrophage erythropoietin signaling results in delayed acute inflammation resolution in diet-induced obese mice
	2009	Li et al	In obesity and type 2 diabetes, elevated levels of saturated fatty acids and reduced levels of omega-3 fatty acids are related to decreased macrophage efferocytosis
	2010	Khanna et al	Macrophage dysfunction impairs resolution of inflammation in the wounds of diabetic mice
Liver injury	2018	Bukong et al	Abnormal neutrophil traps and inhibited efferocytosis lead to liver injury and sepsis severity after binge alcohol use
	2018	Triantafyllou et al	MerTK expressing hepatic macrophages augments the resolution of inflammation in acute liver failure
Cardiac Dysfunction	2017	Pulanco et al	Complement protein C1q enhances macrophage foam cell survival and efferocytosis
	2017	DeBerge et al	MERTK cleavage on resident cardiac macrophages compromises repair after myocardial ischemia reperfusion injury
	2019	de Couto et al	Cardiosphere-derived cell exposure induces sustained MerTK expression in phagocytic capacity through extracellular vesicle transfer of microRNA-26a (*via* suppression of *Adam17*)
	2013	Wan et al	Enhanced efferocytosis of apoptotic cardiomyocytes through myeloid-epithelial reproductive tyrosine kinase links acute inflammation resolution to cardiac repair after infarction
	2017	Zhang et al	Acute CD47 blockade during ischemic myocardial reperfusion enhances phagocytosis associated cardiac repair
	2017	Nakaya et al	Cardiac myofibroblast engulfment of dead cells facilitates recovery after myocardial infarction
	2017	Doran et al	CAMKIIγ suppresses an efferocytosis pathway in macrophages and up-regulates atherosclerotic plaque necrosis
	2022	Bao et al	Engineered neutrophil apoptotic bodies attenuates myocardial infarction and cardiac function via inducing macrophage efferocytosis and inflammation resolution
	2016	Kojima et al	CD47-blocking antibodies restore phagocytosis and prevent atherosclerosis in multiple mouse models
	2022	Cao et al	Sonodynamic therapy promotes efferocytosis via CD47 down-regulation in advanced atherosclerotic plaque
Cancer	2022	Lin et al	MERTK-mediated efferocytosis promotes immune tolerance and tumor progression in osteosarcoma through enhancing M2 polarization and programmed death ligand-1 expression
	2022	Zhou et al	Blockade of phagocytic receptor MERTK on tumor-associated macrophages augments tumor immunogenicity and potentiates anti-tumor immunity via inducing tumor-cGas and host-STING-dependent type I interferon response

Abbreviations: Dendritic cells, DCs; Gas6, growth arrest specific 6; RA, rheumatoid arthritis; SLE, systemic lupus erythema; ALI, acute lung injury; MFG-E8, milk fat globule-EGF factor 8; ARDS, acute respiratory distress syndrome; ALPS, autoimmune lymphoproliferative syndrome; MERTK, MER proto-oncogene tyrosine kinase; CaMKIIγ, Ca2+/calmodulin-dependent protein kinase γ; NETs, neutrophil extracellular traps; AMPK, AMP-activated protein kinase; HMGB1, high mobility group box-1 protein; BAL, bronchoalveolar lavage; SARS-CoV-2, severe acute respiratory syndrome coronavirus 2.

### Infection

Pathogen-mediated cell death allows the host to limit pathogen multiplication and dissemination: the host’s defense cells engulf and eliminate bacteria through phagocytosis, while they eliminate apoptotic cells through efferocytosis (Figure 2) (Karaji and Sattentau, 2017). However, some pathogens may invade the phagocyte *via* efferocytosis and accelerate their own multiplication and spread by expressing the “tolerate me” signal CD47 on the surface of infected cells, which prevents the cells from being efferocytosed (Brown and Neher, 2012).

A recent study showed that low-virulence strains of *Mycobacterium tuberculosis* stimulated apoptosis, generating apoptotic cells that could be engulfed by phagocytes. *Mycobacterium tuberculosis* remained alive in the phagosome and lysosome-phagosome fusion, further promoting bacteria killing (Tzelepis et al., 2015). Stimulating efferocytosis actually exacerbated *Mycobacterium tuberculosis* infection in mice and blocked the engulfment of apoptotic *Mycobacterium tuberculosis*-infected macrophages (Andersson et al., 2020), and knocking out TIM4 in mice induced defective bacterial growth, suggesting that blockade of efferocytosis couldn’t neutralize bacteria (Divangahi et al., 2009; Nishi et al., 2014). Taken together, *Mycobacterium tuberculosis* infection drives cell necrosis, blocks the uptake of infected apoptotic cells by macrophages, and prevents efferocytosis, leading to bacterial spread (Tzelepis et al., 2015).

*Klebsiella pneumoniae* infection can inhibit apoptosis and trigger non-apoptotic programmed cell death mechanisms such as necroptosis (Jondle et al., 2018). At the same time, it can prevent the efferocytotic engulfment of neutrophils by macrophages in the lungs (Codo et al., 2018; Jondle et al., 2018). *Klebsiella pneumoniae* infection was noted to be associated with increased activity of PS transporter flippases and reduced PS externalization and caspase activity (Birge et al., 2016). Blockade of necroptosis restored the efferocytotic ingestion of *Klebsiella pneumoniae*-infected neutrophils (Jondle et al., 2018).

Macrophages phagocytose influenza A virus-infected HeLa cells in a phosphatidylserine-dependent manner during the process of cellular apoptosis. Moreover, engulfment of influenza A virus-treated cells resulted in suppression of virus growth. It was shown that influenza A virus-infected cells appeared to be susceptible to macrophage phagocytosis (Shiratsuchi et al., 2000; Watanabe et al., 2002; Lim et al., 2020). Efferocytotic engulfment of apoptotic HIV-1-infected cells by astrocytes in the brain increases resistance to infection and reduces viral spread (Larsson et al., 2002; Werfel and Cook, 2018). Recently, it was observed that severe acute respiratory syndrome coronavirus 2 (SARS-CoV-2) infection could cause widespread cell apoptosis (Chan et al., 2020) and boost the release of chemokines and cytokines (Dutta et al., 2022). The “cytokine storm” was potent to impair macrophage function and impedes efferocytosis of apoptotic cells (dos-Santos et al., 2021). In contrast, the human papilloma virus suppresses efferocytosis, thus exacerbating infection (Bosnjak et al., 2005). Nevertheless, only a few studies on the effects of efferocytosis on viral spread have been reported.

Engulfment of *Trypanosoma cruzi*-infected apoptotic T cells by macrophages accelerated parasite expansion and promoted the production of TGF-β and PGE_2_, thereby improving virus infectivity (Decote-Ricardo et al., 2017). Efferocytosis is inhibited in sepsis through unknown mechanisms, and blockade of apoptotic cell efferocytosis by phagocytes exacerbates sepsis by increasing levels of sepsis-associated histones and DAMPs that impair apoptotic cell ingestion (Bukong et al., 2018). The extracellular cold-inducible RNA-binding protein (cCIRP), a DAMP that can initiate inflammatory response, has been recently identified. cCIRP-primed NETs prevented efferocytosis in a mouse sepsis model by reducing the levels of integrins αvβ3/αvβ5 in macrophages, indicating that targeting cCIRP *via* the efferocytotic pathway may be a new therapeutic approach against septic challenge (Cohen et al., 2016).

### Acute Lung Injury

ALI is characterized by sustained inflammatory response, disruption of the endothelial-epithelial barrier, alveolar injury, and pulmonary edema, and its pathogenesis has been associated with impaired efferocytosis in a mouse model (McCubbrey and Curtis, 2013). Alveolar macrophages help maintain lung homeostasis during ALI by rapidly removing apoptotic neutrophils through efferocytosis and exerting antimicrobial activity. Moreover, M1-type macrophages induce the expression of STAT6 and relieve ALI by triggering the expression of Gas6, an efferocytotic ligand (Shibata et al., 2020). A recent study indicated that pentraxin 3 located on the membrane of apoptotic cells facilitated macrophage efferocytosis efficiently and alleviated lung inflammation in hard metal-induced acute lung injury (Yan et al., 2022).

NETs are critical for immobilizing and preventing pathogen invasion by releasing pro-inflammatory cytokines and proteases (Jorgensen et al., 2016). However, the increased formation of NETs along with their incomplete efferocytotic uptake may exacerbate inflammation in the development of ALI (Grégoire et al., 2018). Interestingly, restoration of AMP-activated protein kinase activity by metformin or blockade of HMGB1 in bronchoalveolar lavage fluid promoted NETs efferocytosis, and it might provide a potential therapeutic target for attenuating persistent lung inflammation in ALI (Fox et al., 2010).

### Asthma

Asthma is characterized by hyper-responsiveness and exaggerated inflammatory cell infiltration in the airways. Airway allergens can induce the production of inflammatory cytokines by tissue-resident mast cells and facilitate eosinophil migration to the airways (McCubbrey and Curtis, 2013). Defective efferocytotic ingestion of apoptotic cells by airway macrophages has been associated with the pathogenesis of asthma: the excessive accumulation of dying cells stimulates sustained inflammation and secondary necrosis in the lungs (Juncadella et al., 2013). Hence, restoring efferocytosis may be a promising therapeutic approach to eliminate inflammation in asthma. For instance, the release of galectin-3 by macrophages significantly augmented phagocytosis, chemotaxis, and cell activation. However, galectin-3 levels in the sputum of asthma patients were low, thus impairing efferocytosis and allowing sustained airway inflammation (Erriah et al., 2019). Galectin-3 stimulates efferocytotic engulfment of apoptotic cells by airway macrophages in asthma, suggesting that elevated galectin-3 levels might be a way to rescue efferocytosis in asthma (Erriah et al., 2019).

### Systemic Lupus Erythematosus and Arthritis

SLE is a common autoimmune disease with various clinical symptoms manifesting in the lung, heart, kidney, joint, skin, and nervous system, and its pathogenesis has recently been associated with abnormal efferocytosis (Abdolmaleki et al., 2018). The complement factor C1q binds to apoptotic cells *via* IgM and LPC signals and its deficiency may contribute to the development of SLE. Impairment of C1q in mice lacking the efferocytotic bridging molecule MFG-E8 markedly reduced the uptake of apoptotic cells and necrosis in response to autoantibodies and cellular compartments (Hanayama et al., 2004; Hu et al., 2009; Pulanco et al., 2017). By allowing the aggregation of unengulfed dying cells, C1q deficiency contributes to the development of SLE-associated glomerulonephritis. Indeed, genetic targeting of the complement C1q subcomponent subunit A increases the number of apoptotic cells and favors the generation of autoantibodies and glomerulonephritis (Potter et al., 2003).

RA is a chronic inflammatory and progressive joint disorder manifested by the production of serum autoantibodies against rheumatoid factor, complement protein C3, and citrullinated peptides (Abdolmaleki et al., 2018). Given that DNA is degraded by dnase II in lysosomes, dnase II deficiencies have been associated with the pathogenesis of RA and polyarthritis, which are lysosomal storage diseases (Martin et al., 2014). Aggregated DNA in macrophage lysosomes of dnase II-deficient mice activated innate immune response, but other undigested cellular constituents in the lysosomes stimulated production of TNF-α and interferon-β (Waterborg et al., 2018).

These results clearly support that aberrant efferocytosis can disrupt self-tolerance and contribute to the development of several autoimmune disorders. However, further studies are needed to explore the underlying mechanisms.

### Autoimmune Lymphoproliferative Syndrome

ALPS is characterized by increased numbers of CD4^−^CD8^−^ T cells, high levels of circulating IL-10 and Fas ligand (FasL), and hypergammaglobulinemia (Abdolmaleki et al., 2018). The Fas/FasL pathway is crucial for cell apoptosis and its mutation has been observed in a mouse model of ALPS. The TNF receptor family is involved in apoptosis and helps limit the accumulation of self-reactive T and B lymphocytes. Therefore, impaired apoptosis stimulates the immune system due to FasL mutation and defective signaling (Bleesing et al., 2001; Hao et al., 2008), while the Fas/FasL cascade may act as a “find me” signal during efferocytosis (Cullen et al., 2013).

### Multiple Sclerosis

MS is a chronic degenerative disease of the central nervous system characterized by axonal injury, demyelination, oligodendroglial cell death, persistent inflammation (Abdolmaleki et al., 2018), and excitotoxicity and activation of metabotropic (P2Y) as well as ionotropic (P2X) receptors and ATP *via* the glutamate pathway [94]. P2Y and P2X have been found to recognize “find me” and “eat me” signals in MS, implicating dysregulated efferocytosis in the disease (North, 2002; Locovei et al., 2007). The link between efferocytosis and pathogens further needs to be clarified.

### Diabetes

Pancreatic B cell destruction leads to hyperglycemia and insulin deficiency, and it has been closely related to the pathogenesis of diabetes, especially the type I form. Moreover, insufficient removal of apoptotic B cells results in aggregation of dying cells, which in turn mediates the release of autoantigens and the activation of inflammatory signals. Studies in mouse models had linked abnormal efferocytosis with diabetes (Morioka et al., 2019; Doran et al., 2020).

Abnormal efferocytosis in obesity, which is a key factor in type II diabetes, also contributes to defective erythropoietin (EPO) signaling. Specifically, S1P produced in apoptotic cells can bind to the cognate receptor on macrophages, promoting efferocytosis *via* the EPO-EPO receptor-peroxisome proliferator-activated receptor-γ signal (Luo et al., 2016; Luo et al., 2019).

A study in mice showed that low-density lipoprotein receptor deficiency resulted in lesional efferocytosis and to larger necrotic cores than those in healthy animals (Li et al., 2009). Incomplete efferocytosis can slow wound healing and allow persistent inflammation due to apoptotic cell accumulation at the wound site (Khanna et al., 2010), which are the most common complications in diabetes. Of note, much remains to be clarified concerning how abnormal efferocytosis contributes to diabetes.

### Ulcerative Colitis

Ulcerative colitis (UC) is a chronic form of inflammatory bowel disease, characterized by the accumulation of uncleared apoptotic cells in inflammed tissue. Recent studies have shown that enhanced apoptosis or abnormal efferocytosis contribute to the pathogenesis of UC (Abdolmaleki et al., 2018; Morioka et al., 2019; Doran et al., 2020). Bacterial host recognition is critical to the pathophysiology of UC, as lipopolysaccharide (LPS), the main component of the bacterial cell wall, can bind to TLR4 and activate NF-κB-associated inflammatory cascades (Török et al., 2004). Other inflammatory complexes, such as bacterial permeability-increasing and LPS-binding proteins, are also recognized by CD14 in UC patients (Baird et al., 2016), while interaction of CD14 with intercellular adhesion molecule 3 favors efferocytosis by promoting the recognition and engulfment of dying cells (Weiss, 2003). Engulfment of apoptotic corpses by DCs and epithelial cells controlled the disease in a mouse model (Dejani et al., 2018), confirming that targeting efferocytosis might help reduce gut inflammation.

### Liver Injury

MERTK, a TAM receptor, acts as a key bridging molecule during efferocytosis. TAM receptor-deficient mice were more prone to autoimmune hepatitis-like diseases, while increased MERTK-expressing macrophages that infiltrated into necrotic sites were been observed in patients with acute liver injury (Nguyen et al., 2014; Toda et al., 2014; Bernsmeier et al., 2015; Horst et al., 2019; Rymut et al., 2020). MERTK deficiency in a mouse model of acute liver injury increased numbers of myeloperoxidase (MPO)^+^ neutrophils and reduced numbers of liver macrophages, indicating the critical role of MERTK in the clearance of dying neutrophils (Triantafyllou et al., 2018). In addition, giving the mice with acute liver injury a secretory leukocyte protease inhibitor reduced the number of MPO^+^TUNEL^+^ neutrophils, suggesting a therapeutic approach against acute hepatic damage (Triantafyllou et al., 2018). Patients with decompensated cirrhosis and acute-on-chronic hepatic dysfunction showed an increased number of MERTK-expressing macrophages and monocytes, which linked to reduced levels of the pro-inflammatory mediators IL-6 and TNF-α (Bernsmeier et al., 2015). This clinical finding suggests that administering MERTK inhibitors can enhance the production of these pro-inflammatory cytokines in MERTK^+^ monocytes (Bernsmeier et al., 2015). Thus, expanding the population of MERTK-expressing myeloid cells may promote the removal of necrotic components and thereby serve as a treatment against acute liver injury.

Similar to MERTK, Gas6 is a bridging molecule that is highly expressed in liver macrophages and can be stimulated with carbon tetrachloride (CCI_4_). Gas6 deficiency reduces numbers of infiltrating monocytes and levels of pro-inflammatory mediators, limiting hepatocyte expansion and Kupffer cell proliferation (Nepal et al., 2019). Moreover, Gas6/Ax1 signaling in CCI_4_-induced hepatic injury blocks NLRP3 inflammasome activity and autophagy, which is important to homeostasis maintenance (Shibata et al., 2020). Incomplete neutrophil efferocytosis following excessive alcohol consumption in a mouse model exacerbated sepsis-associated liver injury, revealing a potential therapeutic target against liver injury (Bukong et al., 2018).

### Cardiac Dysfunction

Efferocytosis has been reported to relate to the process of myocardial repair (Yoshimura et al., 2020). Deleting MERTK from macrophages in mice with reperfusion injury impaired cardiac function, increased infarct size, and reduced cardiac wound debridement (DeBerge et al., 2017). Conversely, delivery of MERTK and C1q to macrophages *via* extracellular vesicles enhanced efferocytosis and cardioprotection in mice after myocardial infarction (Wan et al., 2013; de Couto et al., 2019). Likewise, MFG-E8 deficiency leads to cardiac inflammation, necrosis, and cardiac dysfunction (Soki et al., 2014), whereas MFG-E8 administration has the opposite effects (Zhang et al., 2017). Cardiac myofibroblasts producing MEF-E8 can efficiently recognize dead cardiac cells and thereby promote recovery from myocardial infarction (Nakaya et al., 2017). Interestingly, a recent study showed that engineered neutrophil apoptotic bodies attenuated myocardial infarction and cardiac function via inducing macrophage efferocytosis and inflammation resolution (Bao et al., 2021).

Inflammatory signaling in apoptotic cells at lesion sites results in overexpression of the “tolerate me” signal CD47, promoting resistance to internalization and thereby compromising efferocytosis (Willingham et al., 2012). Treatment with a neutralizing anti-CD47 antibody during ischemic myocardial reperfusion accelerated the removal of apoptotic myocytes by phagocytes, promoted the resolution of cardiac inflammation, preserved cardiac function, and reduced infarct size, implying that targeting CD47 might protect against myocardial reperfusion (Kojima et al., 2016; Cao et al., 2022). Mesenchymal stem cells in a rat model were reported to improve cardiac function by promoting M2 macrophage-mediated efferocytosis of apoptotic neutrophils (Doran et al., 2017).

These results illustrate the close association between efferocytosis and inflammatory resolution in cardiac disorders. Notably, they demonstrate the potential for exploiting efferocytosis to attenuate myocardial ischemia-reperfusion injury and control immune response.

### Cancer

Tumor-associated macrophages act as a type of phagocyte involved in efferocytosis. These macrophages are M2-polarized and promotes the production of anti-inflammatory mediators and regulatory T cells, suppressing effector T cells. Subsequently, removal of dying cells by macrophages inhibits inflammatory responses and provides tumor cells a microenvironment to escape from immunological surveillance (Soki et al., 2014). It has been demonstrated that efferocytosis not only facilitates the proliferation, invasion, metastasis, and angiogenesis of tumor cells, but also affects the drug resistance to anti-cancer treatments (Birge et al., 2016; Barkal et al., 2018; Barkal et al., 2019; Tajbakhsh et al., 2021). Recently, Lin et al. reported that MERTK-mediated efferocytosis promotes immune tolerance and tumor progression in osteosarcoma through enhancing M2 polarization and programmed death ligand-1 expression (Lin et al., 2022). Furthermore, an excellent study revealed that blockade of phagocytic receptor MERTK on tumor-associated macrophages augmented tumor immunogenicity and potentiated anti-tumor immunity via inducing tumor-cGas and host-STING-dependent type I interferon response (Zhou et al., 2020). Thus, efferocytosis-targeted therapy may represent a potential approach for treating cancers.

## Conclusion and Perspectives

Phagocytes rapidly remove apoptotic cells *via* efferocytosis to ensure tissue repair and organ development. Efferocytosis involves the recognition of “find me” and “eat me” signals, followed by phagosome-lysosome fusion and digestion of apoptotic corpses. Phagocytes selectively recognize and ingest apoptotic cells because normal cells display “tolerate me” signals. However, it is unclear whether the “tolerate me” and “eat me” signals are cell-specific and whether they exert different impacts on phagocytic cells in certain circumstances.

In this review, we focus on the potential role of efferocytosis in the regulation of immune response and homeostasis, and the effect of aberrant efferocytosis on the pathogenesis of inflammatory disorders. Macrophage efferocytosis of apoptotic cells stimulates the differentiation of macrophages into a pro-resolving phenotype by enhancing the production of pro-resolving mediators and angiogenic growth factors and by reducing the levels of pro-inflammatory cytokines. In this way, efferocytosis prevents an excessive inflammatory response and favors tissue repair. On the one hand, efferocytosis regulates immune response, such as by eliminating invading pathogens; on the other hand, some pathogens can “hijack” efferocytosis to infect phagocytes, where they multiply and spread in a protected manner *via* a “Trojan horse” mechanism.

The importance of efferocytosis is reflected in the fact that defects in the process contribute to various inflammatory disorders. Understanding more about what regulates efferocytosis and developing ways to activate it may be useful therapeutic approaches against inflammatory diseases. Most investigations of efferocytosis have been performed in cellular and animal studies, with little development being made in the clinical context. Further research is critically needed to explore the impact of regulating efferocytosis on susceptibility to inflammatory pathologies and the safety of this approach in clinical settings. Additionally, it is possible that there is a dynamic balance of efferocytosis involved in inflammation and immunity or tissue repair. Accordingly, studies are warranted to precisely evaluate the function of efferocytosis and immune responses in inflammatory disorders Weirather et al., 2014.
